# Breast cancer in Iraq is associated with a unimodally distributed predominance of luminal type B over luminal type A surrogates from young to old age

**DOI:** 10.1186/s12905-017-0376-0

**Published:** 2017-04-07

**Authors:** Runnak A Majid, Hemin A Hassan, Dana N Muhealdeen, Hazha A Mohammed, Michael D Hughson

**Affiliations:** 1 Department of Pathology, Shorsh General Hospital and the Hiwa Regional Oncology Center, Qirga Road, 46001 Sulaimaniyah, Kurdistan, Iraq; 2 Department of Oncology, Hiwa Regional Oncology Center, Sulaimaniyah, Kurdistan, Iraq

**Keywords:** Breast cancer, Iraq, Cancer incidence, Breast cancer receptors, Luminal A and luminal B breast cancer, Middle-East

## Abstract

**Background:**

Breast cancer has recently increased in post-menopausal Iraqi women. In Western countries at high-risk for breast cancer, there is a bimodal increase in estrogen receptor (ER) positive tumors with a peak of low proliferation rate luminal A over higher proliferation rate luminal B tumors after 60 years of age. The aim of this study was to analyze in Iraqi women whether shifts are occurring in immunohistochemical (IHC) surrogates of molecular breast cancer subtypes toward a high-risk profile.

**Methods:**

Age specific and age standardized womens breast cancer incidence was estimated for the years 2006 through 2012. IHC results of ER, PR, HER2, and Ki67 testing were analyzed on the breast cancers of 125 Arabic and 725 Kurdish women by frequency of distribution and by age.

**Results:**

Between 2006 and 2012, age standardized incidence of breast cancer in Iraq increased from 30 to 40/100,000 women with the increase specifically occurring in women ≥ 60 years old (*P* < 0.001). Breast cancers in Kurdish women ≥ 60 years old may also have increased (*P* = 0.047) with urban exceeding rural rates by 2:1. For both Kurdish and Arabic women, there was a marked predominance of luminal B tumors at all ages in which luminal B and luminal A tumors were asymmetric skewed toward older age but with no late luminal A age peak.

**Conclusions:**

Older Iraqi women do not show the bimodal shift toward higher rates of luminal A breast cancers seen in the West. The modest increase in age standardized incidence of breast cancer in Iraqi is being seen specifically in older women and may be better attributed to a trend for care in urban cancer centers rather than changing tumor characteristics.

## Background

In the developing world, cancer is projected to increase by 70% over the next 20–25 years. Breast cancer represents 20–30% of cancer among women and is likely to account for a major part of that increase [1]. These expectations are anticipated because the populations of developing countries are ageing and cancer is largely a disease of older people. Life-style changes are likely to contribute, because transitions from a traditional to a Westernized life-style expose women to higher individual risk [2].

The world can be divided into low-risk and high-risk regions on the basis of differences in breast cancer incidence [1, 2]. Those at high-risk consists of the developed countries of Northern Europe, North American, Uruguay, Argentina, Australia and New Zealand. In these areas, age standardized incidence exceeds 80-100/100,000 women. In the developing world, age standardized incidence (ASI) varies from 20-60/100,000 women, and in parts of Central and Southeast Asia and sub-Saharan Africa, estimates are less than 20/100,000 [1, 2].

Breast cancer in all parts of the world begins to be seen at about 20 years of age [3]. Afterward, age specific incidence steadily rises until the menopause when geographic differences begin to be seen. In the developing world, a flattening and then a decline occur about 10–15 years after the menopause. In developed countries, the age specific incidence accelerates after the menopause, and at age 70 and above doubles that seen at 45–49 years old [3–5]. The life-style changes contributing to increased post-menopausal breast cancer are largely premenopausal and include obesity, low rates of childbirth, infrequent or no lactation, early menarche, and late menopause [2]. These factors, that are common in high-risk countries, promote a state of relative hyperestrogenism and the development of estrogen responsive tumors [3–5]. The life-style factors are becoming more common in countries considered low-risk particularly in their growing urbanized communities [6].

Molecular subtyping has defined at least four categories of breast cancer with the most prevalent type and that being most closely related to increased estrogen exposure being estrogen receptor (ER) positive and negative for the Her-2-neu (HER2) proto-oncogene [3]. ER positive tumors are further subtyped into low proliferation rate luminal A and higher proliferation rate luminal B tumors with the luminal A tumors being more frequently seen in older women in regions of the world at high-risk for breast cancer [3].

Breast cancer rates in Iraq and its Kurdish region were generally stable between 2000 to 2009, but newer data from the Iraqi Cancer Registry reveal rising rates since 2009 with women after age 50 making the major contribution to the increase [5, 7–9]. In this study, demographic changes are explored together with tumor ER and HER2 subtyping. The aim of the study was to determine whether age related shifts may be occurring in ER and HER2 subtypes that may offer insight into potential increases of breast cancers in culturally transitional Middle-Eastern countries.

## Methods

### Calculation of age specific and age standardized incidence

Data for the country of Iraq were obtained from the publications of the Iraqi Cancer Registry for the years 2006 and 2012 [9, 10]. These publications provided detailed information regarding the age specific incidence rates of breast cancer and the female population of Iraq in the 5-year age ranges from 0 to 70+ years old. The proportions of the population in the 5 year age ranges correspond to that published by the United Nations Population Division (population pyramid.net).

Data for Sulaimaniyah were obtained from the Hiwa Regional Oncology Center cancer registry. The data collection was limited to the years before 2014 so as to not be influenced by the large recent influx of internally displaced persons into the Kurdish region. Breast cancers for Sulaimaniyah were averaged for the three year periods 2006–2008 and 2011–2013. The 2006 and 2012 Sulaimaniyah populations were obtained from the Iraqi Cancer Registry and matched with the population estimates from the Sulaimaniyah Department of Statistics. The 5 year age distribution followed the proportions used for all of Iraq.

For each region, age specific incidence rates were calculated on the basis of the number of cancers per 100,000 women in the specified age ranges. Age standardized incidence rates were calculated using the WHO World Standard population.

### Analysis of breast cancer subtypes

Immunohistochemistry (IHC) for ER, progesterone (PR), HER2, Ki67, and HER2 FISH for IHC equivocal HER2 cases was performed for breast cancers of Hiwa Hospital patients at the Shorsh Hospital Pathology Department. Details of the technical procedures have been previously published [5]. The luminal A subtype was defined as ER+/HER2- with a KI67 index ≤20% and PR+ with an Allred PR score ≥ 4; the luminal B subtype was defined as an ER+ and/or PR+ tumor, ± HER2+, and with a Ki67 index > 20% [11–14].

### Statistical methods

Data was entered into an Excel worksheet and analyzed with Stata IC10™ (Stata Corp, College Station, TX) and SigmaStat 3.5 (Systat Software Inc., San Jose, CA) statistical software. Changes in breast cancer incidence in the years 2001 to 2012 were analyzed by Poisson’s regression for the age ranges 20–39, 40–49, 50–59, and 60+ years old [15]. Differences between groups were tested by Kruskall-Wallace analysis of variance (ANOVA) on ranks with Dunn’s post-hoc pairwise tests. Age by ethnicity was normally distributed for some receptor subtypes but not others, and two-way comparisons used Wilcoxon rank-sum tests. For all statistical procedures *P* < 0.05 was considered significant.

## Results

### Age distribution of Iraqi and Sulaimaniyah populations

The Sulaimaniyah population estimates and incidence rates are for ethnic Kurdish women only. Table 1 specifies in 5-year age ranges the raw data for female populations and the numbers of women’s breast cancers for the years 2001, 2006, and 2012 for Iraq. There is no data available for Sulaimaniyah in 2001, and only 2006 and 2012 are shown.

**Table 1:** The womens population and the numbers of breast cancers in Sulaimaniyah and all of Iraq by 5 year age groups

	Sulaimaniyah				Iraq					
	2006		2012		2001		2006		2012	
	pop	BC	pop	BC	pop	BC	pop	BC	pop	BC
0–4	129187	0	136092	0	1843227	0	2352412	0	2365695	0
5–9	120025	0	126440	0	1586575	0	1995955	0	2192043	0
10–14	110863	0	116788	0	1423251	0	1721195	2	2024941	0
15–19	98036	0	103276	0	1259927	5	1530129	6	1800966	1
20–24	84292	1	88798	1	1119935	14	1312396	18	1551925	26
25–29	69633	4	73355	4	956611	66	1125539	55	1282305	76
30–34	61387	11	64668	14	816619	121	953623	136	1129203	203
35–39	55890	21	58877	19	583300	244	794087	272	1026864	355
40–44	48560	28	51155	41	466640	334	638097	388	884658	610
45–49	40314	30	42469	37	419976	386	514935	412	738741	677
50–54	28403	28	29921	28	279984	345	399765	441	522486	582
55–59	21989	26	23165	26	233320	201	310221	262	403572	481
60–64	19241	11	20269	18	209988	175	228969	214	358341	426
65–69	10995	11	11582	12	193324	107	166953	141	205681	252
70+	16492	9	17373	15	256652	122	272958	155	300103	333

Legend: The breast cancers for Sulaimaniyah represent the averages for the three year periods 2006–2008 and 2010–2013. For Iraq, the numbers are for the indicated year. The year is designated by the availability of population data within the period that the breast cancers were enumerated. Abbreviations: *Pop*,population, *BC* breast cancer

### Age standardized and age specific incidence

Table 2 demonstrates the age specific incidence rates in the regions and the changes over the period from 2001 to 2012. Prior to 2012, the age standardized rates were approximately 30 per 100,000 women with a peak age specific incidence at 50–54 years old and a declining rate in older age groups. At 70+ years old, rates were similar to those seen at 35–39 years of age. In all of Iraq in 2012, age specific incidence exceeded 110/100,000 for all 5-year age groups from 50 to 70+ years old, and the age standardized rate increased to 40/100,000. In Sulaimaniyah, age standardized incidence increased slightly from 32 to 36/100,000 between 2006 and 2012.

**Table 2:** Age specific incidence per 100,000 women in five-year age groups for Sulaimaniyah and all of Iraq for the indicated years

	Sulaimaniyah		Iraq		
Age (yrs)	2006	2012	2001	2006	2012
0–4	0	0	0	0	0
5–9	0	0	0	0	0
10–14	0	0	0	0	0
15–19	0	0	0	0	0
20–24	1	2	1	1	2
25–29	6	6	7	5	6
30–34	18	22	15	14	18
35–39	38	32	42	34	35
40–44	57	81	72	61	69
45–49	74	88	92	80	92
50–54	99	95	123	110	111
55–59	117	113	86	85	119
60–64	56	89	83	94	119
65–69	103	103	66	85	123
70+	56	84	48	57	111
Total rate	32	36	33	32	40

Legend: Rates are rounded to the nearest whole number. The total rate is age standardized using the WHO world standard population (2002)

### Distribution of breast cancer by age

By Poissons regression, in all of Iraq over the decade 2001–2012, there was a significantly increased breast cancer rate between 2006 and 2012 among women ≥ 60 years old (P <0.001) but not in younger age groups (Table 3). Table 3 also indicates that for Sulaimaniyah, breast cancer rates may also have increased in the ≥ 60 year old age group (*P* = 0.047).

**Table 3:** Poisson regression analysis of the changes in age specific incidence rates in the indicated age ranges over the period from 2001 to 2012 in all of Iraq and over the period 2006 to 2012 for Sulaimaniyah

	Sulaimaniyah		Iraq	
Age (years)	coefficient	*P*	coefficient	*P*
20–39	0.000 (−0.148 to 0.148)	1.000	0.000 (−0.078 to 0.078)	1.000
40–49	0.051 (−0.013 to 0.116)	0.12	−0.003 (−0.034 to 0.029)	0. 87
50–59	−0.008 (−0.062 to 0.046)	0.78	0.008 (−0.018 to 0.035)	0.54
60+	0.064 (0.001 to 0.128)	0.047	0.063 (0.032 to 0.094)	<0.001

### Rural vs urban differences

We attempted to look at urban vs rural differences in breast cancer rates. Data from Baghdad were not available. In 2011–2013, 63.1% of Sulaimaniyah breast cancer patients identified themselves as living in the central city (3-year average, central city 137 patients vs other governate 80 patients). In 2012, the central city of Sulaimaniyah was estimated to have a population of 867,000 (433,500 women) (www.theodora.com/wfbcurrent/iraq/iraq_people.html). This would provide a crude incidence of 31.6/100,000 for the central city and 15.0/100,000 for women from the outlying areas of the Governate.

### Breast cancer subtypes by age and ethnicity

**Fig. 1:**
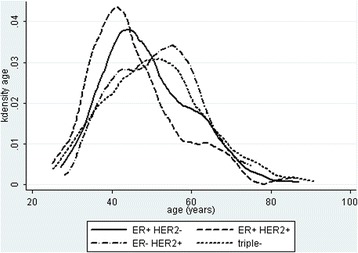
Kernal density plots for the four major ER and HER2 subtypes Legend: ER+/HER2+ tumors peak at a significantly earlier age than ER+/HER2- tumors (*P* < 0.01). There is no significant difference in the age distribution of other subtypes (ANOVA, *P* > 0.05)

**Fig. 2:**
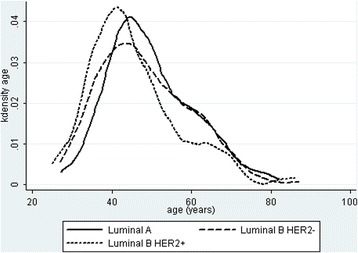
Kernal density plots of ER+ tumors separated into luminal B HER2+, luminal B HER-, and luminal A tumors Legend: Luminal B/HER2+ tumors developed first at a significantly earlier age than luminal A tumors (*P* < 0.01). Luminal B/HER2- were found next, but the age distribution was not significantly different than for luminal A tumors (*P* = 0.19)

In the years 2011–2013, 850 primary breast cancers from Hiwa Hospital patients (725 Kurdish and 125 Arabic) were analyzed. The average age of Kurdish patients was 48.9 ± 11.9 and Arabic patients 49.3 ± 11.5 years, a not significant difference (*P* = 0.72). For Kurdish and Arabic patients combined, the distribution by ER+/HER2-, ER+/HER2+, ER-/HER2+, triple negative, and luminal A and luminal B subtypes in the age ranges 20–39, 40–49, 50–59, and 60+ years old are shown in Table 4. The great majority of tumors were ER+/HER2- (64.8%) but with the luminal A cancers representing just 27.4% and luminal B nearly twice that number at 46.8% of subtypes. Triple negative comprised 12.8% and ER-/HER2+ 13.0% of tumors. Figures 1 and 2 are kernel density plots by age for the major subtypes. Figure 1 reveals an earlier onset of ER+/HER2+ (luminal A/HER+) and triple negative compared to ER+/HER2- and ER-/HER2+ tumors. ER+/HER2- tumors showed a pronounced skew into older age, but all subtypes overlapped with no significant differences in age between patients with ER+/HER2-, ER-/HER2+, and triple negative tumors (ANOVA, *P* > 0.05). The proportional diagnoses of receptor types by Kurdish or Arab ethnicity were not significantly different (ER+/HER2-, 457 vs 85; ER+/HER2+, 68 vs 11; ER-/HER2+, 98 vs 11; triple-, 90 vs 17; *X* ^2^ = 2.40, *P* = 0.49), and ethnicity versus age was not statistically different for any receptor type (Table 5).

**Table 4:** Sulaimaniyah ER and HER2 breast cancer subtypes by age groups, Kurdish and Arabic

Age (years)	ER+/HER2-	ER+/HER2+	ER-/HER2+	Triple-	Luminal A	Luminal B
20–39	97	19	30	28	23	67
40–49	189	33	30	31	64	96
50–59	146	18	27	26	39	57
60+	110	9	22	22	30	48
All (%)	542 (64.8)	79 (9.4)	109 (13.0)	107 (12.8)	156 (27.4)*	268 (46.8)*
Ave age	49.7 ± 11.7	46.2 ± 11.0^a^	47.3 ± 10.4	48.8 ± 13.2	49.7 ± 10.7^b^	47.6 ± 11.3
Ki67	26.1 ± 18.6 ^c^	39.9 ± 21.0	43.1 ± 23.0	56.6 ± 26.5 ^c^	11.6 ± 5.0	37.9 ± 18.4

Legend: *Luminal A and luminal B are derived from ER+/HER2- and ER+/HER2+ tumors that had Ki67 testing with the percentages being extrapolated to the sum of all subtypes. The number of luminal A and Luminal B tumors (*n* = 424) does not correspond to the number of ER+/HER2- and ER+/HER2+ tumors (*n* = 621), because not all of the later had Ki67 testing. The age of ER+/HER2+ patients is significantly different than ER+/HER2- patients (*P* < 0.01)^a^. The age of luminal A patients is significantly different than luminal B patients (*P* = 0.04)^b^. The Ki67 of ER+/HER2- and triple- breast cancers are significantly different than other subtypes (*P* < 0.001)^c^

**Table 5:** Comparison of Arab and Kurdish ethnicity versus age for the specified breast cancer receptor types

Receptor type	Arab (*n*)	Kurdish (*n*)	*P*
ER+/HER2-	48.3 ± 10.5 (85)	49.0 ± 11.1 (457)	0.83
ER+/HER2+	48.7 ± 10.5 (11)	44.9 ± 10.8 (79)	0.61
ER-/HER2+	51.2 ± 7.8 (11)	1.2 ± 10.9 (98)	0.91
Triple-	53.7 ± 18.2 (17)	50.7 ± 12.1 (90)	0.81
Luminal A	51.7 ± 11.9 (15)	49.5 ± 10.6 (141)	0.53
Luminal B	46.8 ± 11.2 (31)	47.7 ± 11.4 (237)	0.66

Legend: Values are displayed as mean ± standard deviation. Comparisons are made by Wilcoxon rank sum tests. The receptor subtypes by age were not significantly different between Kurdish or Arabic women

### Age and ethnic distribution of luminal A and luminal B tumors

**Fig. 3:**
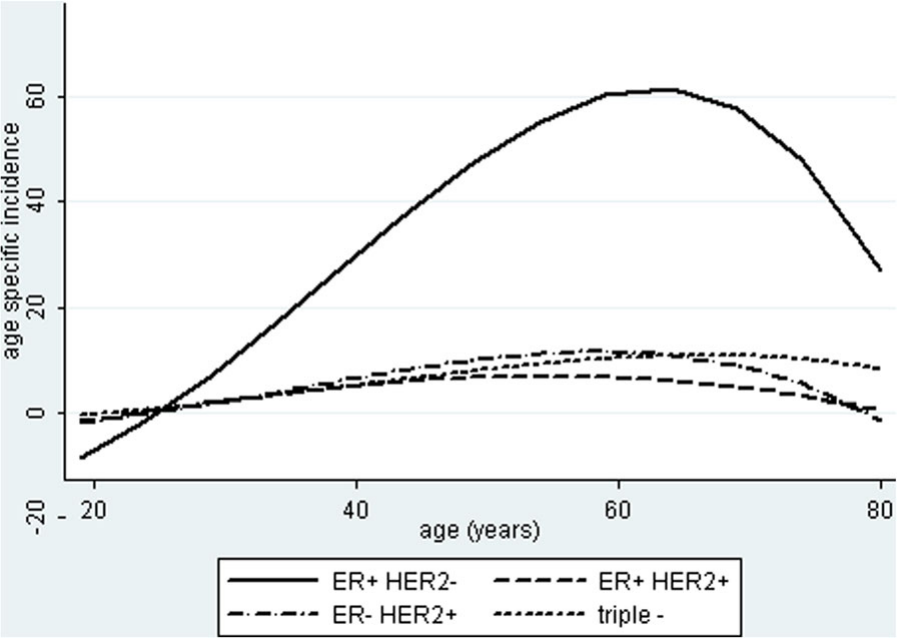
Age specific incidence of the four major ER and HER2 subtypes Legend: Polynomial regression plots show a peak incidence predominantly of ER+/HER2- tumors at approximately age 55–60 years that rapidly declines afterward

**Fig. 4:**
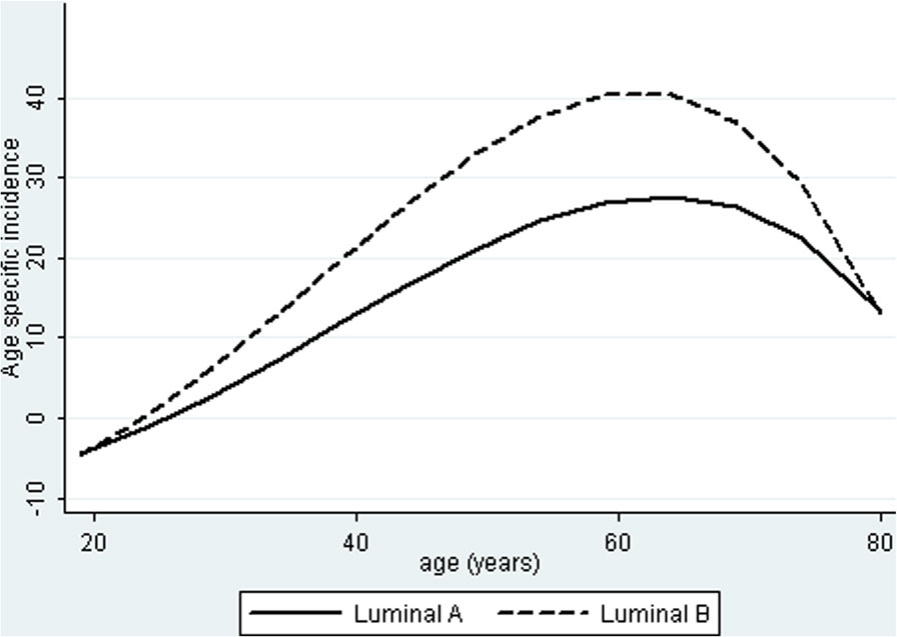
Age specific incidence of luminal A and luminal B tumors Legend: Polynomial regression plots showing a predominance of luminal B at all ages over luminal A tumors. There is no late luminal A peak that exceeds the incidence of luminal B tumors

Figure 2 shows that luminal B/HER2- tumors began to be seen at an earlier age than Luminal A tumors, but both subtypes were closely nested over the entire range of young to old age with no suggestion of a late luminal A peak. Luminal B/HER2+ tumors had a significantly earlier peak than the other subtypes (*P* < 0.01). Although Luminal B/HER2+ tumors represented only 28% of all luminal B tumors, the earlier age contributed to all luminal B tumors being found at a significantly earlier age than luminal A tumors (*P* = 0.04) but with no significant difference in age between luminal A and luminal B/HER2- tumors (*P* = 0.19). Figure 3 plots age specific incidence per 100,000 women for the major subtypes. The peak incidence of all cancers was about 60 years of age but then declined markedly from 60 to 80 years old. The decline includes luminal A and luminal B subtypes with the incidence of luminal B exceeding that of luminal A at all ages (Fig. 4).

## Discussion

Molecular breast cancer subtyping is widely used clinically to identify patients at low risk for metastasis and can be roughly inferred on the basis of IHC testing [12, 13]. While the 2015 St Gallen’s consensus considered the IHC designation of molecular subtypes as being impractical for guiding treatment, the assignment may be valuable from an epidemiologic standpoint [3, 13]. In the West, the average age of newly diagnosed breast cancer patients is > 60 years old, and the high rates of breast cancer are the result of a several-fold increase in the age specific incidence of ER+/HER2- tumors in post-menopausal women [3–5].

In US women, on the basis of IHC with parallel PAM50 molecular testing, luminal B breast cancers were found to be more common than luminal A tumors by a factor of 2.48 under 40 years of age with the ratio decreasing to 1.27 at 40–49 years old [16]. At 50–59 years of age, the proportions reached parity, and at > 60 years old, luminal A exceeded luminal B tumors by 25-50% [16]. In terms of age specific density, this created a bimodal frequency for ER+ tumors in which there is a minor peak mainly of luminal B tumors at about age 50 and a major peak due to the late contribution of luminal A tumors near age 70 [3].

This is not seen in Sulaimaniyah for Kurdish or Arab breast cancer patients where the average is < 50 years old for both ethnic groups. In these Iraqi patients, luminal B tumors were the predominant subtype from the beginning of breast cancer development in the early 20s and remained predominant at all later ages. The age distribution of both luminal A and luminal B tumors was unimodal and closely overlapping with no suggestion of a late peak for ER+ tumors.

On the basis of both molecular testing and IHC surrogates, this greater proportion of luminal B over luminal A breast cancers has been observed in other low incidence countries, including Colombia [age standardized incidence (ASI), 39.7] and China (ASI, 31.7) [17–19]. In reports of Middle-Eastern patients in which luminal A were distinguished from ER+/PR+ luminal B tumors on the basis of a low Ki67 index, a > 3:1 predominance of luminal B over luminal A was found in Saudi Arabia (ASI, 24.7) and in Moroccan Arab immigrants to Belgium [20, 21]. In the Belgium study, Moroccan and European women were compared [21]. The mean age of Moroccan women was 49 ± 11 years old. European women who averaged 60 years old showed a contrasting all age parity in the proportion of luminal A and B tumors.

A 2013 report from Jordan showed that despite an increasing incidence of breast cancer in older women, the median age of all Jordanian breast cancer patients was still 50 years old [22]. The authors claimed a predominance of luminal A tumors, but the cancers were not identified as luminal A based upon the St. Gallen’s guidelines and included all luminal subtypes except those that were HER2 positive [22].

The 2013 St Galen’s guidelines recommended that luminal A be defined as ER+/PR+/HER2- tumors with a Ki67 index ≤ 14% and PR expression in > 20% of cells [12]. Higher proliferation rate ER+ tumors including the triple positive ER+/PR+/HER2+ subtype are considered luminal B. The 2015 guidelines suggested a Ki67 index ≤ 20% or a Ki67 index less than the laboratory average for ER+/HER2- tumors [13]. Regardless of the specific Ki67 criteria, a low proliferation rate is needed to reasonably replicate a luminal A molecular phenotype.

In Sulaimaniyah patients, triple negative and ER-/HER2+ were nearly evenly distributed throughout all age groups at a frequency approximately half that of luminal A tumors. This is representative of triple negative and ER-/HER2+ subtypes in other low incidence countries and illustrates their relatively low contribution to the total breast cancer burden compared to luminal tumors [5, 18, 19, 23, 24]. Triple negative tumors are unusually common in sub-Saharan and African American women [3, 24, 25]. However, in other racial groups they seem to have a relatively constant frequency of about 10-15% and increase proportionately across all ages as the general rate of breast cancer rises [3, 5, 17–19, 24–26].

The relatively higher rates of luminal B breast cancers in developing countries may be a reflection of the younger populations and the relatively small proportions of aging women exposed to premenopausal hyperestrogenism. We have previously reported that the age specific incidence of the common ER+/HER2- breast cancer is similar in Kurdish and US women under 50 years old [5]. The difference in the two populations is the absence in Kurdistan of the marked increase in the rates of ER+/HER2- tumors in older women that is seen in the US [5]. This lends credibility to a concept of a prolonged population exposure being needed for the development of luminal A tumors. Nevertheless, it is worth mentioning that the age of the population by itself is not the determining factor. China has a huge, aging population but a low breast cancer age standardized rate and low rates of luminal A tumors [17, 23]. In addition, low parity, that is considered a risk factor﻿ for post-menopausal tumors in the West, has been public policy in China for decades.

Our findings show that breast cancer incidence was essentially the same in the Kurdish region and in the south of Iraq before 2010 with an age standardized rate of approximately 30 per 100,000 women. After 2009, rates increased to 40.0/100,000 women in the south of Iraq and with a smaller change in Sulaimaniyah. This seems to be following the pattern observed over the past decade in Jordan (ASI, 53.8), Lebanon (ASI, 55.4), and several Gulf States including Kuwait (ASI, 48.0) and Qatar (ASI, 45.0) and may be an early phase in the transition toward higher breast cancer rates [2].

It is not obvious whether the increasing age standardized rate of breast cancer in Iraq and other Middle-Eastern countries is a true biological transition or whether it reflects the increased utilization of urban cancer centers [5, 19, 26, 27]. With a more general awareness of breast cancer, financially able women may selectively be traveling for a level of care that is not within the means of many families. In 2011, 60.1% of breast cancer among Jordanian women was diagnosed in Amman where the crude incidence rate was 47.8/100,000 [28]. This compared with the outlying Governates where crude incidence varied from 11.7 to 22.7/100,000 women. In the Sulaimaniyah Governate, a similar clustering of breast cancer diagnosis was found in central Sulaimaniyah city. This may indicate urban/rural differences in incidence but as likely represents patterns of accessing cancer care.

The technology and specialization needed for modern cancer care necessitates its centralization but also has the potential for creating demographic artifacts. With mammography and MRI, central facilities have the ability to detect small and in-situ breast cancer that may not be found by clinical practice outside of major centers. In addition, women taking advantage of these facilities may be more likely to have risk factors associated with post-menopausal disease.

In many if not most Middle-East countries, luminal subtypes represent more than 60% of breast cancers of all ages and more than 70% after age 60 [5, 6, 20–22, 26, 27]. Whether the pattern of increasing rates of breast cancer in older patients is taking a direction that will resemble the West remains to be seen, and it will be valuable to understand the relationships between cancer subtypes in the region. If the Western pattern is followed, age standardized rate should continue to rise, the average age of breast cancer patients should increase, and luminal A should exceed the frequency of luminal B tumors at around 60–70 years of age.

## Conclusions

Older Iraqi women do not show the bimodal shift toward higher rates of luminal A breast cancers seen in the West. The modestly increased breast cancer rates being seen in some Middle-Eastern countries may be the result of factors different from those occurring in the West and may not represent a transition to high-risk breast cancer rates. As an epidemiological study, the major limitation is that the design is observational and retrospective and that breast cancer subtyping is lacking in Iraq outside of the Kurdish region.

## Abbreviations

ANOVA: Analysis of variance
ASI: Age standardized incidence
ER: Estrogen receptor
HER2: Her-2-neu
IHC: Immunohistochemistry
PR: Progesterone receptor
